# Breaking down walls

**DOI:** 10.7554/eLife.42033

**Published:** 2018-10-24

**Authors:** Moagi Tube Shaku, Bavesh Davandra Kana

**Affiliations:** 1 DST/NRF Centre of Excellence for Biomedical TB Research, Faculty of Health SciencesUniversity of the Witwatersrand and the National Health Laboratory ServiceJohannesburgSouth Africa

**Keywords:** tuberculosis, antibiotics, Mycobacteria, cell wall, peptidoglycan, sidewall, Other

## Abstract

A better understanding of the mechanisms underpinning the growth of mycobacteria could help identify targets for new antibiotics.

**Related research article** García-Heredia A, Pohane AA, Melzer ES, Carr CR, Fiolek TJ, Rundell SR, Chuin Lim H, Wagner JC, Morita YS, Swarts BM, Siegrist MS. 2018. Peptidoglycan precursor synthesis along the sidewall of pole-growing mycobacteria. *eLife* **7**:e37243. doi: 10.7554/eLife.37243

**Related research article** Baranowski C, Welsh MA, Sham LT, Eskandarian HA, Lim HC, Kieser KJ, Wagner JC, McKinney J, Fantner GE, Ioerger TR, Walker S, Bernhardt TG, Rubin EJ, Rego EH. 2018. Maturing *Mycobacterium smegmatis* peptidoglycan requires non-canonical crosslinks to maintain shape. *eLife* **7**:e37516. doi: 10.7554/eLife.37516

The rise of antimicrobial resistance has significantly reduced the options available for treating tuberculosis (TB) and other diseases caused by bacteria, threatening to plunge humanity back into the pre-antibiotic era. *Mycobacterium tuberculosis* is a rod-shaped bacterium that is responsible for around 10 million new infections and 1.4 million deaths per year (Pai et al., 2016). With an estimated 0.5 million cases of drug-resistant TB every year, this disease now accounts for a substantial fraction of the global burden of antimicrobial resistance (WHO, 2018). The current treatment for drug-resistant TB involves taking a combination of antibiotics over a period of up to 24 months, or six months for drug-sensitive TB. Such extensive treatment periods, with associated side effects, including deafness, create an urgent need for new drugs that shorten the duration of treatment, reduce the daily pill burden, and are effective against drug-resistant strains.

As the bacterial cell wall has formed the basis of successful antibiotic therapy for decades, the search for new TB drugs has led to renewed interest in the mycobacterial cell wall as a drug target (Alderwick et al., 2015). Mycobacteria, a genus that also includes the bacteria that cause leprosy and Buruli ulcer, have a complex cell wall that consists of an outer capsule-like layer, anchored by long-chain fatty acids known as mycolic acids, and two polymers (Kieser and Rubin, 2014). One of these polymers is called arabinogalactan; the other one, peptidoglycan, is made from monomers that contain a sugar molecule and a short peptide chain that consists of three to five amino acids. The sugar molecules bind together to form a long chain that is further strengthened by the formation of crosslinks between neighboring peptide chains; mainly between the third amino acids on both peptide chains (to form a 3–3 crosslink), or between the fourth amino acid of one and the third amino acid of the other (to form a 4–3 crosslink) (Raghavendra et al., 2018).

Current tuberculosis drugs that target the cell wall affect the synthesis of the mycolic acids and the arabinogalactan layer, but none are directed at peptidoglycan. Paradoxically, this polymer (in particular the cross-linking process) has been a successful drug target in other bacterial infections, but treating tuberculosis with the same drugs has so far been fruitless. This is mainly due to inherent mechanisms in *M. tuberculosis* that inactivate certain types of antibiotics, and to the fact that we know relatively little about how this polymer is synthesized and remodeled as the bacteria spread (Story-Roller and Lamichhane, 2018). Now, in eLife, two independent groups of researchers report results that advance our understanding of these processes (García-Heredia et al., 2018; Baranowski et al., 2018).

As bacteria grow and divide, peptidoglycan needs to be constantly broken down and reassembled. In other rod-shaped bacteria, such as *Escherichia coli*, growth is achieved by the insertion of new peptidoglycan units along the sidewall. However, the way mycobacteria grow is very different. Their growth happens at the polar regions (that is, at each end of the bacterium) through the addition of new units of peptidoglycan to the sub-polar region of the cell wall. Mycobacteria further lack homologues of *E. coli* proteins that direct peptidoglycan growth to the sidewall. Consequently, in mycobacteria, rapidly changing peptidoglycan segregates to the cell poles, while inactive peptidoglycan remains at the sidewall (Figure 1). However, preliminary observations have suggested an active sidewall peptidoglycan 'metabolism', but definitive evidence of this phenomenon, together with a detailed mechanistic explanation, has been lacking.

**Figure 1.**
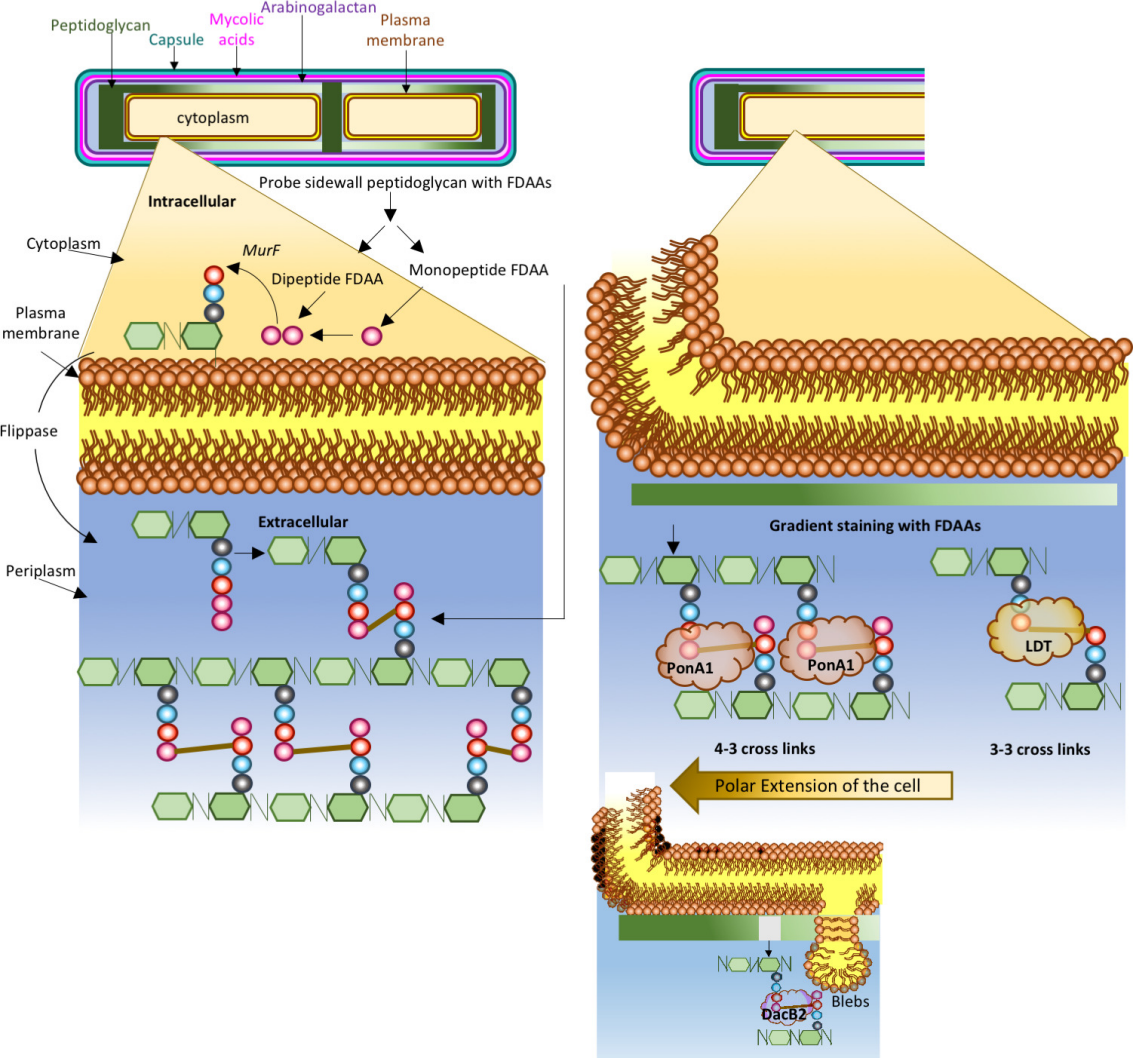
The synthesis and remodeling of peptidoglycan in mycobacteria during growth. The cell wall of a mycobacterium consists of an outer capsule-like layer (teal line), mycolic acids (magenta line), arabinogalactan (purple line), peptidoglycan (green lines) and a bilayer of fatty acids comprising the cell membrane (orange line and circles with tails). These protect the cytoplasm, which is surrounded by the periplasm. Peptidoglycan is made from monomers that contain a sugar molecule (green hexagon) and a chain of three to five amino acids (different colored circles). Precursor peptidoglycan units are synthesized inside the cell and linked to a lipid carrier. These units are transported into the periplasm by an enzyme called a flippase. Crosslinks (brown lines) between the monomers lead to the formation of a rigid matrix that encases the cell. Garcia-Heredia et al. (left) used monopeptide and dipeptide FDAA probes (one and two pink circles respectively) to study both the synthesis of new peptidoglycan and the remodeling of existing peptidoglycan: the monopeptide probes can be incorporated into peptidoglycan (in the place of the amino acid D-Alanine) both inside and outside the cell, whereas the dipeptide probes can only be incorporated inside the cell. Previously, it was thought that growth in mycobacteria only happened at the poles, where peptidoglycan is active. However, the work of Garcia-Heredia et al. suggests that there is a gradient of peptidoglycan activity along the sidewall of the dividing mycobacterium, with a maximum (dark green) near the poles and a minimum (pale green) near the center, and that the synthesis and the remodeling can both occur at the sidewall. Baranowski et al. (right) confirm that the side wall contains active peptidoglycan. Moreover, they show that the crosslinks change from the 4–3 confirmation at the poles to the 3–3 confirmation at the sidewall. The 3–3 crosslinks are formed by enzymes called L,D-transpeptidases (LDTs); deleting these enzymes affects the activity of DacB2, an endopeptidase that can break down peptidoglycan, which leads to the formation of blebs (see inset) and damage to the cell wall. The 4–3 crosslinks are formed by an enzyme called PonA1, which is a D,D-transpeptidase. Taken together, these two studies provide a revised paradigm for peptidoglycan remodeling in mycobacteria that could help with the development of new drugs to target the mycobacteria that cause diseases such as tuberculosis.

Sloan Siegrist of the University of Massachusetts and colleagues – including Alam García-Heredia and Amol Arunrao Pohane as joint first authors – report the results of experiments on *M. tuberculosis* and its close relative, *M. smegmatis*, where they explore peptidoglycan biology further (García-Heredia et al., 2018). The team used reagents called fluorescent D-amino acids (FDAAs) to monitor the arrival and distribution of new peptidoglycan units both inside and outside the cell, as well as the remodeling of peptidoglycan outside the cell (Figure 1). They found that the proteins involved in the synthesis of new peptidoglycan units and remodeling of existing polymers can be found at both cell poles and at the sidewall. The amount of polymer incorporated into the sidewall decreased gradually from the poles to the middle of the cell. The cells only grew from the poles, suggesting that the remodeling process in the sidewalls may have a different purpose.

García-Heredia et al. then used antibiotics to either block the synthesis of new peptidoglycan units or the remodeling process, and found that both processes affected the incorporation of FDAAs into the cell wall differently. This indicated that peptidoglycan precursors are produced and remodeled along the sidewall of mycobacteria. Moreover, a group of enzymes called L,D-transpeptidases appeared to play a crucial role during peptidoglycan remodeling or repair when the bacterium responded to peptidoglycan damage. To back this up further, FDAAs marking the new production sites were predominantly incorporated at the sidewall when the bacteria were exposed to peptidoglycan-damaging antimicrobials.

In independent work, Hesper Rego of Yale, Eric Rubin of Harvard and colleagues – including Catherine Baranowski as first author – report results on how the structural properties of peptidoglycan change as the polymer ages during bacterial growth (Baranowski et al., 2018). Mycobacteria distinguish themselves from other bacteria by having a large proportion of 3–3 crosslinks in their peptidoglycan, whereas bacteria such as *E. coli* tend to have a very high proportion of 4–3 crosslinks.

Baranowski et al. also used FDAAs and found that the amount of probe incorporated into the sidewall decreased from the cell poles to the middle of the cell, as García-Heredia et al. had reported. However, when they removed all L,D-transpeptidases, the enzymes that drive the formation of 3–3 crosslinks, FDAA uptake was reduced, the bacteria lost their rod shape, and bulges called 'blebs' started to appear in the cell wall. Atomic force microscopy then revealed that the cell wall at a bleb was weaker than it was elsewhere.

Baranowski et al. propose that peptidoglycan at the cell pole most likely consists of 4–3 crosslinks. However, as the cell grows, peptidoglycan ages and moves to the sidewall, where it is remodeled into the 3–3 conformation (Figure 1). Consistent with this, D,D-transpeptidases, the enzymes that help to form 4–3 crosslinks, preferred to stay at the cell pole, while L,D-transpeptidases were predominantly found on the sidewall.

These observations confirm that the sidewall of the mycobacteria undergoes continuous restructuring, a process that possibly renews aged peptidoglycan. Moreover, the remodeling process relies on the careful coordination and distinct spatial patterning of various proteins, including the enzymes for the formation of 4–3 crosslinks and 3–3 crosslinks. The change from the 4–3 to the 3–3 confirmation is needed to increase the strength of peptidoglycan in the side wall and to maintain the rod shape of cells as they grow.

Collectively, the two studies provide unique mechanistic insights into the metabolism of peptidoglycan and how mycobacteria coordinate their growth. FDAA markers in combination with some of the genetic tools used provide an ideal starting point from which to develop screens for candidate drugs that target peptidoglycan biosynthesis and remodeling. Further study of these processes will undoubtedly drive the identification of new antimicrobials that could meaningfully add to the existing arsenal of tuberculosis drugs. As peptidoglycan can be found in many pathogenic bacteria, the new drugs that emerge from such efforts might also be able to treat other infectious diseases.
